# New perspective on targeting the tumor suppressor p53 pathway in the tumor microenvironment to enhance the efficacy of immunotherapy

**DOI:** 10.1186/s40425-015-0053-5

**Published:** 2015-03-24

**Authors:** Gang Guo, Yan Cui

**Affiliations:** Department of Biochemistry and Molecular Biology, Cancer Immunology, Inflammation & Tolerance Program, Georgia Regents University Cancer Center, Augusta, GA 30912 USA

**Keywords:** Cancer, Tumor suppressor p53, *p53* inactivation, Tumor microenvironment, Immune suppression, Inflammation, Antitumor immunity, Immunotherapy

## Abstract

About 50% of human cancers harbor somatic mutations of the *tumor suppressor p53* (*p53* or *Trp53*) gene. Many of those mutations result in the inactivation of the p53 pathway and are often associated with the stabilization and accumulation of mutant p53 proteins. Therefore, increased p53 expression in tumors is frequently used as a surrogate marker for p53 mutation and inactivation. Moreover, this elevated p53 expression also makes it an ideal tumor associated antigen (TAA) for cancer vaccines. Recent advances in our understanding of p53 as a crucial transcription factor reveal that p53 is an important sensor of cellular stress under genotoxic, chemotoxic, pathological, and even normal physiological conditions. Experimental and clinical observations by our laboratory and others have demonstrated that p53 also participates in immune regulation as p53 dysfunction skews host immune responses towards pro-inflammation, which further promotes tumor progression. Furthermore, recent studies using a genetic approach revealed that p53-restoration or re-activation led to tumor regression and clearance, which were at least partially caused by the activation of innate antitumor immunity. Since many of the currently used cancer therapeutics, including radiotherapy and chemotherapy, disrupt tumor growth by inducing DNA damage via genotoxic or chemotoxic stress, which activates the p53 pathway in the tumor microenvironment, we postulate that some of those observed therapeutic benefits might also be partially mediated through their immune stimulatory effects. Here, we briefly review our current understanding of the potential cellular and molecular mechanisms by which p53 participates in immune regulation and, subsequently, extend our discussion to the immunostimulatory potential of existing and new approaches of targeting the p53-pathway to alter the immunological landscape of tumors for maximizing immunotherapy outcome.

## Introduction

The *tumor suppressor p53* (also called *transformation related protein 53*, *Trp53*), was first described in 1979 as an oncogene, but was subsequently cloned and characterized as a tumor suppressor gene in its wild-type configuration in 1989 [1-4]. It is now known that *p53* encodes a crucial transcription factor controlling the life and death of a cell and is the most frequently mutated gene in tumors [5-7]. Generally, about 50% of human tumors harbor *p53* mutations, mostly missense, although the frequency varies among different tumor types [5-7]. These mutations often cause conformational changes of the p53 protein, which consequently impairs its DNA binding capacity, resulting in loss of p53 function and reduced sensitivity to apoptosis or senescence, a permanent status of irreversible cell cycle arrest [7-9]. Furthermore, these conformational changes often stabilize the p53 protein resulting in an elevated p53 level in tumors, which is frequently used as a surrogate marker of p53 mutation [5-7,9]. Experimental and clinical evidence suggests that both mutant and wild-type p53 are immunogenic because anti-p53 antibodies and p53 antigen-specific T cells are detected in tumor patients [10-12]. Thus, both forms of p53 have been employed as tumor associated antigens (TAAs) in tumor vaccine clinical trials [13-15].

Even though *p53* is the best studied gene as the result of 30 years extensive research, our comprehension and appreciation of its complexity in regulating many crucial biological processes are far from complete [4,9,16-18]. Immunologically, besides using p53 as a TAA, whether *p53* mutation and/or dysfunction imposes immunological consequences of promoting tumorigenesis has largely been unexplored. Numerous experimental and clinical results demonstrate that environmentally induced damage and genetic instability are associated with *p53* dysfunction and inflammation [19-21]. Now that chronic inflammation is a well-accepted hallmark of cancer [22-24], it is plausible that *p53* dysfunction may also contribute immunologically to tumorigenesis and tumor progression by altering host immune responses. In fact, recent results from our laboratory and others have demonstrated that *p53* dysfunction skews tumor milieu towards pro-tumor inflammation [25-27], whereas *p53* reactivation or restoration reverses the immunological landscape towards antitumor immunity [28-30]. Thus, it will be important for us to comprehend the mechanism of p53 activation-induced antitumor immunity and appreciate the unintended immunological components of conventional non-immunotherapy regimens that activate the p53 pathway. As the focus of this perspective review is on the involvement of p53 in immune modulation, we only present a brief and simplified view of the cellular and molecular pathways mediating p53 regulation and function. Subsequently, we extend our review and elaborative discussion to the immunological aspect of p53 function. We will conclude with new perspectives on future applications of maximizing antitumor efficacy by combining therapies targeting the p53 pathway with active immunotherapy.

## Review

### Trp53 - the master regulator of stress response and its canonical mechanism of tumor suppression

Trp53 is a master transcription factor that regulates the expression of a plethora of genes involved in crucial biological processes, many of which encode proteins that control the cell cycle or induce apoptosis [7,8,31]. Because of its critical impact on cell fate, cellular p53 activity must be precisely controlled. Usually, p53 is ubiquitously expressed in almost all cell types, but is barely detectable under normal physiological conditions in unstressed cells [7,8]. This low basal p53 level is controlled and regulated by its inhibitor molecules, MDM2 (mouse double minute 2 homolog) and MDM4 (also called MDMX). MDM2, an E3 ubiquitin ligase and the major regulator of p53 stability and activity, promotes the rapid degradation of p53 and prevents it from binding to the promoters of p53 target genes [32,33].

**Figure 1:**
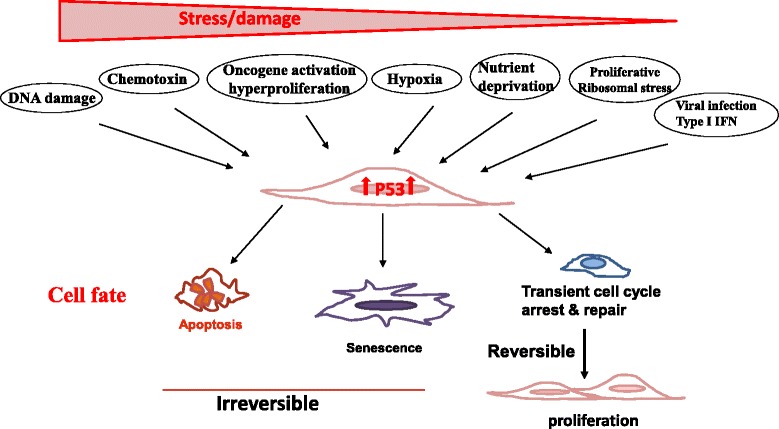
**Trp53 is a crucial sensor of cellular stress and a guardian of the genome.** The tumor suppressor p53 is ubiquitously expressed in almost all cell types but is barely detectable under physiological conditions in unstressed cells. When a cell incurs various environmental or endogenous stresses, such as DNA damage, chemotoxin, oncogene activation, hypoxia, nutrient deprivation, replicative ribosomal stress, and viral infection, cellular p53 is activated causing an elevated level of p53 protein associated with its acetylation and phosphorylation. Activated p53 subsequently transactivates multiple molecular pathways, which induce apoptosis, senescence (a permanent non-reversible cell cycle arrest), or transient cell cycle arrest, depending on the level and nature of the stress, as well as the severity and reversibility of the damage that cells incur. Whereas severe stress and irreparable damage lead to apoptosis or senescence, modest stress and repairable damage cause transient cell cycle arrest for repair. The cell will re-enter the cell cycle to produce progeny once the damage is repaired. Thus, p53 plays an important role in ensuring proper health and function of all cells and is regarded as the caretaker, gatekeeper, and guardian of the genome.

When a cell incurs DNA damage by genotoxic, chemotoxic stress, or receives aberrant signals from oncogene activation, p53 is activated causing an elevated level of p53 associated with its acetylation and phosphorylation (Figure 1) [7-9]. These post-translational modifications of p53 prevent its sequestration by MDM2, leading to its increased stability [32,33]. Activated p53 subsequently transactivates multiple molecular pathways, which induce cell cycle arrest and/or senescence via upregulating p21, the cyclin-dependent kinase inhibitor 1, and apoptosis via promoting puma (p53 upregulated modulator of apoptosis), noxa, or bax (Bcl2-assocated X protein).

Recent emerging evidence reveals that p53 can also be activated by various physiological and pathological stressors, including hypoxia, ribosomal stress, endoplasmic reticulum (ER) stress, metabolic stress, nutrient deprivation, viral infection, and psychological stress [9,16,17] (Figure 1). Thus, as a crucial sensor of cellular stress, p53 plays an important role in ensuring proper health and function of all cells by dictating their fate of apoptosis, senescence, or transient cell cycle arrest, depending on the level and nature of the stress as well as the severity and reversibility of the damage that cells incur (Figure 1) [7,8,34-37]. Though severe stress and unrepairable damage leads to apoptosis or senescence, modest stress and repairable damage causes transient cell cycle arrest. Cells will re-enter the cell cycle to produce progeny once the damage is repaired (Figure 1). This well regulated induction of apoptosis and senescence is considered the major mechanism by which p53 suppresses tumor development and ensures genome stability. Therefore, p53 is regarded as the caretaker, gatekeeper, and guardian of the genome [7,8,31].

### Non-canonical and non-cell autonomous mechanisms of p53 mediated tumor suppression

In the past decade, compelling evidence reveals that p53 participates in regulating a wide array of biological processes throughout the entire lifespan of the organism [4,9,16,18,38-40]. Thus, it is not surprising that *p53* dysfunction may result in dysregulation of many biological processes, such as stem cell homeostasis, metabolism, autophagy, angiogenesis, migration, and invasion [4,17,41-43], all of which are linked to the hallmarks of cancer [23,44].

For instance, one of the hallmarks is the altered metabolic pathway in cancerous cells to glycolysis as the predominant source of ATP production, the so-called Warburg effect [45]. Recent studies demonstrated that p53 suppresses glycolysis in three ways: (1) reducing Glut3 expression and, thus, suppressing glucose uptake; (2) enhancing the expression of mitochondrial respiratory enzyme SCO2 (synthesis of cytochrome c oxidase); and (3) promoting the expression of TIGAR (TP53-induced glycolysis and apoptosis regulator) [35,46-48]. Thus, *p53* inactivation results in a switch to glycolysis by enhancing glucose uptake and reducing SCO2 and TIGAR. These observations shed light onto the molecular mechanism by which the Warburg effect in cancers can be caused by *p53* inactivation/dysfunction. Other studies also revealed that p53 activation promotes autophagy either directly via DRAM (damage regulated autophagy mediator) or indirectly via the AMP-activated protein kinase (AMPK) and the mTOR pathway [49-51]. In addition, p53 is also shown to suppress cell invasion by repressing the NF-kB mediated podia intrusion/formation and to inhibit epithelial-to-mesenchymal transition (EMT) by suppressing the expression of SNAIL and ZEB (members of zinc-finger transcription factors) [42,52-54]. Therefore, the tumor suppressive function of p53 is partially mediated by altering cellular metabolism, motility, and invasion [55,56], which are considered non-canonical mechanisms.

**Figure 2:**
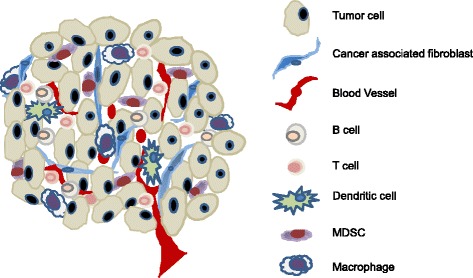
**Cellular constituents of the tumor microenvironment that shape the immunological landscape of tumor.** The tumor microenvironment consists of complex molecular and cellular constituents. Tumor regression or progression is dictated by its immunological landscape, i.e. activated antitumor immunity or tumor-induced immune tolerance/immunosuppression, which is reflected by the activation status of T and B lymphocytes, NK cells, macrophages, dendritic cells, and other myeloid derived cells. Importantly, the immunological landscape is also greatly impacted by cells of the non-hematopoietic compartment, including cancer associated fibroblasts (CAFs) and endothelial cells of the lymphatic and blood vasculature. Moreover, *p53* inactivation has been reported to occur in various cells within the tumor microenvironment, such as CAFs, which further promotes immunosuppression and augmented tumor progression. Therefore, targeted activation of the p53 pathway to enhance antitumor immunity should not limited to tumor cells, but extend to cellular compartments of the CAFs and potentially immune cells as well.

In addition to the aforementioned direct effects on tumor cells, p53 also suppresses tumorigenesis via changing the function and property of cells adjunct to tumors, such as cancer associated fibroblasts (CAFs). Clinical and experimental evidence confirms the existence of somatic *p53* mutations in CAFs and highly inflamed pathological tissues [57-60]. Moreover, *p53* mutations in CAFs of breast and prostate cancer patients, whose cancer cells maintain wild-type p53 function, were associated with an increased rate of metastasis and poor prognosis [5,58,59,61]. These results suggest that *p53* dysfunction in CAFs serves as a selective pressure for the transformation of adjacent epithelial cells [62]. This tumor suppressive effect of p53 via altering the milieu of transformed cells is regarded as the non-cell-autonomous mechanism [57,62]. Accumulating evidence suggests that tumor progression and metastases are markedly affected by the molecular and cellular constituents surrounding the tumors, the so-called tumor microenvironment (TME) [63-65]. It is now greatly appreciated that both the cellular and molecular components of the TME are highly complex. The cellular compartment consists of immune cells of T-, B-, NK-, and myeloid cells and non-hematopoietic stromal cells, including CAFs, lymphatic, and blood endothelial cells (Figure 2). The molecular components that greatly impact tumor progression include integrins and other extracellular matrix proteins, cytokines, and chemokines. Mechanistic studies revealed that the pro-tumor effect of *p53*-dysfunctional CAFs is mediated through enhanced production of cytokines and chemokines, including SDF-1 and IL-6, which further affected immune cell composition and function within the TME [25,57,66,67]. Together, these observations showed that *p53* dysfunction in tumors or other populations within the TME also promotes tumor progression and metastasis via mechanisms including, but not limited to, the suppression of apoptosis and/or senescence.

### Immunosuppressive effects of *p53* dysfunction on the host immunological landscape

Compelling evidence demonstrates that chronic inflammation plays a vital role in tumor initiation, progression, and metastasis [20,23,24,68,69]. Nevertheless, it has not been explored systemically whether p53 participates in immune regulation or whether *p53* inactivation causes chronic inflammation, further promoting tumorigenesis and tumor progression.

Interestingly, it was observed as early as the 1980s that p53 expression was elevated in mitogen-stimulated or T cell receptor (TCR)-ligation activated T cells [70,71]. Subsequent experiments and clinical observations further confirmed the activation or alteration of the p53 pathway during immune responses and in inflamed pathological tissues; however, the immunological significance of p53 activation remains unknown [72-75]. Based on the observations that *p53* inactivation exacerbated autoimmune disease in mice [76,77], we and others proposed that p53 suppresses autoimmunity by inhibiting inflammation [20,21,26,76]. Specifically, Zheng et al. showed that *p53* inactivation enhanced the production of inflammatory cytokines IL-1, −6, and −12 by macrophages [76] while we demonstrated that the genetic deletion of *p53* enhanced IL-6-induced Th17 differentiation and promoted the spontaneous development of autoimmunity in *p53*^*null*^*CD45.1* mice [26]. Because Th17/IL-17 activity has been linked to inflammation, autoimmunity, and tumorigenesis [78-80], these observations further support the role of *p53* inactivation in inflammation-induced tumorigenesis.

Importantly, this *p53* inactivation mediated elevation of inflammatory molecules is not restricted to immune cells because *p53* mutations/inactivation in CAFs also augment their production of pro-inflammatory molecules [25,58,59,61], which is associated with increased metastasis and poor prognosis [66,81,82]. It is noteworthy that in cells maintaining wild-type *p53*, functional inactivation of the p53 pathway can also be instigated by other means, such as overexpression of the p53 inhibitor MDM2 or the viral proteins HPV E6 and HLTV-1 Tax, all of which have been linked to tumorigenesis [83-88].

Mechanistically, it has been suggested that p53 activity has an inverse correlation with that of NF-κB, a major transcription regulator of inflammatory response [21,89,90]. It is believed that the reciprocal activities of the p53 and NF-κB are the result of their competition for the limited transcription coactivator p300 and the CREB-binding protein [21,89,90]. Recent studies also demonstrated that p53 inhibits NF-κB-dependent genes by directly suppressing the promoter activity of NF-κB subunit p65 or indirectly repressing the activity of IKKα, a subunit of IκBα kinase complex [48,90,91]. Thus, p53 suppresses the expression of IL-6, Cox-2, and iNOS, and thereby inflammation, by inhibiting NF-κB activity [20,21,89]. Likewise, *p53* inactivation causes the hyperactivity of NF-κB in *p53*^*null*^ T cells, macrophages, and intestinal epithelium, leading to chronic inflammation and tumorigenesis as shown by us and others [26,76,91,92].

It is noteworthy that the reciprocal activity of the p53 and NF-κB is not exclusive because the co-activation of p53 and NF-κB was also observed under certain circumstances [21,93]. For instance, Lowe and colleagues demonstrated that p53 and NF-κB co-regulate IL-6 production in human macrophages [93]. Additionally, it has been well established that senescent cells secrete numerous inflammatory cytokines, chemokines, growth factors, and other soluble proteins to the extracellular space, which subsequently activate NF-κB [94,95]. Interestingly, the p53-dependent release of the chromatin protein high-mobility group box 1 (HMGB1) by senescent cells is a major mediator of their secretion of other inflammatory molecules via activation of TLR-4 and NF-κB [95]. Thus, the co-activation of p53 and NF-κB also occurs in senescent cells. Nevertheless, it is not yet fully understood how pro-inflammatory molecules produced by senescent cells promote antitumor immunity in certain conditions but enhance pro-tumorigenic inflammation under other circumstances [30,62,94].

Altogether, these results suggest that p53 may serve as a general suppressor of innate immunity and inflammation. *Trp53* dysfunction in either hematopoietic or non-hematopoietic populations alters the immunological landscape of the host/TME to pro-inflammation, thereby immunologically promoting tumorigenesis and tumor progression.

### New insights into the immunological components of therapeutic interventions that activate the p53 pathway

It is now appreciated that activation of anti-tumor immunity is indispensable for the therapeutic benefits of conventional therapies [96-99]. Recent mechanistic studies illustrated that only those therapies eliciting tumor immunogenic cell death (ICD), such as radiotherapy and some chemotherapy regimens, induce a robust anti-tumor immunity [96-100]. On the other hand, therapies causing non-immunogenic death of tumors fail to stimulate a strong antitumor immunity as they are unable to overcome tumor-induced immune tolerance. So far, the identified molecular processes crucial for ICD include the following: (1) the exposure of ER proteins, such as calreticulin (CRT), at the cell surface; (2) the secretion of ATP to extracellular space, which is frequently associated with autophagy; and (3) the release of HMGB1 to extracellular space [96,99].

Although it is well established that radiotherapy and most of the chemotherapy regimens induce DNA damage via genotoxic and chemotoxic stress, which activate the p53 pathway in cells maintaining functional p53 (Figure 1) [7,8], it is yet to be verified clinically whether p53 activation induced in those therapies contribute to the induction of antitumor immunity. Experimentally, animal studies with p53 re-activation or restoration in either tumors or stroma confirmed that tumor regression and clearance are dependent on senescence-induced antitumor immunity [30,62]. As discussed earlier, senescent cells secret HMGB1, inflammatory cytokines, and chemokines to extracellular space [95]. It is conceivable that p53 activation-induced cellular senescence may subsequently trigger autophagy or ICD of cells in the TME, thereby activating antitumor immunity. Alternatively, it is also possible that direct killing of tumors by activated NK cells as the result of p53-activation alters the immunological milieu of tumors to immune stimulatory despite not meeting all the aforementioned characteristics of ICD. In fact, it has been demonstrated that DNA damage and/or p53 activation, either dependent or independent of each other, upregulate the expression of NKG2D ligand such as ULBP2, greatly enhance NK mediated tumor elimination, and alter the antigen presentation capacity of tumors and stromal cells toward immune-stimulation [101-106]. Subsequently, this leads to the production of type I interferon (IFN), activation of M1 macrophages, enhancement of the antigen presentation capacity of tumors, APCs, and stromal cells, and recruitment of immune cells to the TME, all of which synergistically promote antitumor immunity [97-100,107-111]. Furthermore, Taura et al. and Shatz et al. demonstrated that p53 activation upregulates the expression and function of toll-like receptors (TLR)-3 or −8 in human cancers, lymphocytes, alveolar type I cells, and epithelial cell lines [27,112,113]. Likewise, p53 also interacts with IFN regulatory factors (IRF), specifically IRF-5 and IRF-9, and IFN-stimulated gene factor 3 (ISGF3) at various phases of anti-viral immunity [114-116], as well as in cancer cells treated with IFN and a DNA damage agent [117,118]. As both TLR ligands and IFN-alpha are potent immune adjuvants that have been employed for cancer immunotherapy, it is also plausible that some of the immunostimulatory effects of conventional therapies that induce p53 activation are mediated through enhancing the TLR and IFN pathways. Certainly, more in-depth studies are necessary before any definitive conclusions are drawn.

Together, these results suggest that p53-activation either in tumors or stroma by some conventional therapies elicits both innate and adaptive anti-tumor immunity via various molecular mechanisms. It has yet to be clarified whether some of the observed immune-stimulatory effects might be mediated through a p53-independant pathway, especially in tumors incurring *p53* mutations. Nevertheless, it is also important and clinically relevant to examine whether immune responses initiated by p53-activation in CAFs that maintain functional *p53* may overcome the unresponsiveness or, even worse, immune tolerance induced by tumors that incur *p53* mutations. Further studies are necessary to clarify the contribution and quality of p53-activation in promoting antitumor immunity for improving the outcome of immunotherapy [96,98,100,109,110,119].

### Therapeutic strategies of targeted activation and/or restoration of the p53 pathway as adjuvant for enhancing antitumor immunity – current status and new perspective

As discussed above, the immunostimulatory properties of radiotherapy and chemotherapy have received increasing appreciation [98-100,107,109-111]. Importantly, some of the cellular and molecular processes that dictate the outcome of immune stimulation vs. tolerance are better comprehended, although still evolving [96,99]. This better understanding provides a mechanistic explanation of why some of the apparently similar therapies are immunostimulatory at times, but immunosuppressive under other circumstances [96-100,120]. It is also noteworthy that high dose systemic radiation or chemotherapy regimens may impose the side effects of inducing mutagenesis and drug resistance in tumors, besides the lymphoid-hematopoietic toxicity to the host [98,102]. Therefore, it is important for us to better define the following: (1) whether the level or duration of p53 activation dictates the immunological outcome of antitumor immunity so that unnecessary damages can be controlled or avoided altogether, and (2) whether the tumor/host *p53* dysfunction, either pre-existing or therapy-induced, may skew antitumor immunity to pro-tumor inflammation. Clarification of these issues will facilitate the development of more effective strategies combining p53-activation induced ICD/senescence with active immunotherapy to maximize therapeutic benefits.

Because p53 mutation/inactivation is one of major causes of cancer, targeting the p53 pathway via viral vector mediated *p53* delivery or small molecule p53-activators has been an important approach for cancer treatment over the past decades and is still rapidly evolving [121-123]. Different from other conventional therapies, these approaches have greatly reduced risk of lymphoid-hematopoietic toxicity and mutagenesis because they do not induce DNA damage. On the other hand, the immunological properties of most of the currently tested p53-activation regimens have not been explored because they were developed preceding our comprehension and appreciation of p53 activation to antitumor immunity. Here, we will focus our review on the approaches directly targeting the p53 pathway, other than the radiotherapy and chemotherapy that we already discussed above, with insights on their potential immunological effects and capacity for promoting antitumor immunity.

#### Viral vector-mediated *p53* re-introduction or restoration gene therapy

Adenoviral delivery of exogenous *p53* (Ad-*p53*) to tumors represents one of the first series of p53 targeted clinical trials for multiple tumor types worldwide [124-126]. Despite demonstrated p53-induced apoptosis, the clinical outcome of Ad-*p53* therapy has been less than satisfactory due to limited intra-tumor delivery. To improve the delivery efficacy and utilize the *p53*-inactivated nature of many tumors, more advanced vector systems have been developed. For instance, the oncolytic adenovirus ONYX-015 is capable of replicating only in *p53*-defective tumors and inducing their apoptosis [127-129]. So far, these viruses showed limited therapeutic efficacy [127-131]. Due to lack of immunological data, we can only speculate that the less than satisfactory efficacy is due to either the limited activation of p53 not sufficient to induce antitumor immunity [124-126] or the oncolytic virus induced tumor death is non-ICD leading to immune tolerance [127-129]. Interestingly, a clinical trial combining Ad-p53 with chemotherapy by Nemunaitis and colleagues showed certain levels of clinical response [130], implying that an elevated p53 activity in the TME, potentially stromal cells that maintain p53 function, subsequently enhanced antitumor efficacy. However, a more definitive conclusion cannot be drawn in the absence of clinical assessment of immune responses and p53 activity associated with this trial.

#### Small molecule based therapy targeting the p53-MDM2 axis

The E3 ubiquitin ligase MDM2 is a crucial p53 inhibitor. Many tumors, especially hematopoietic malignancies, exhibit loss of p53 function as the result of MDM2 amplification while maintaining wild-type *p53* [32,132]. To target this MDM2-amplification induced functional p53 inactivation, small molecule MDM2 antagonists were developed to re-activate the p53 pathway [132,133]. For instance, Nutlin-3, one of the MDM2 antagonists that occupies the p53 interacting pocket of MDM2, enacts anti-tumorigenic and anti-metastatic effects by preventing p53 degradation, thereby selectively inducing apoptosis and/or senescence of tumor cells [133,134]. Similarly, RITA (reactivation of p53 and induction of tumor cell apoptosis), another small molecule that inhibits the binding of HDM2 (human double minus-2, the human analogue of MDM2), also induces tumor specific p53 activation [132,135]. Overall, the therapeutic effects of these pharmacological p53-activators require the existence of wild-type *p53*, as they are ineffective in tumors with mutant *p53*. Although most of the published studies focus on MDM2 inhibition-induced tumor cell apoptosis, a couple of recent observations demonstrated that Nultin-3 also regulates host immune response by modulating the immunological function of dendritic cells or other antigen presenting cells [136,137], making it a potential candidate of immunotherapy adjuvant. To fully appreciate the effects and quality of p53 activation in promoting antitumor immunity, additional studies to address dose-dependent effects of Nultin-3 or RITA in mediating p53 activation and antitumor immunity are necessary. Furthermore, our better understanding of their cellular and molecular mechanisms of immune activation will greatly expand their clinical application for immunotherapy.

#### Restoration of mutant p53 to wild-type configuration and function

Since many p53 mutants are associated with a conformational change that hinders its DNA binding and transactivation capacity, it is rationalized that small molecules that revert the mutant p53 to its wild-type configuration will restore p53 function. Indeed, based on crystallographic structural and computational analyses, PRIMA-1 (p53 reactivation and induction of massive apoptosis-1) and MIRA-3 (mutant p53 reactivation and induction of rapid apoptosis *in vivo*) were developed to convert mutant p53 and restore p53 function leading to effective activation of downstream apoptosis-inducing targets, such as caspase-2, puma, and Bax [138,139]. Again, previous studies on antitumor effects of p53 reactivation via reverting *p53* mutations mainly focused on the non-immunological aspects. With our better understanding of the immunological component of anti-tumor effects of p53 activation, these small molecules will be valuable tools for evaluating the immunological impact of p53 reactivation in various compartments of the TME, such as tumor or CAFs, both have been shown to incur p53 mutations independent of each other. Thus, these molecules will allow us to examine whether p53 reactivation either in tumors or stroma is sufficient to reverse *p53* dysfunction-induced immunosuppression and the level of p53 reactivation required to achieve sufficient antitumor immunity to overcome tumor-induced immune tolerance.

#### Combining p53-activation therapy with active immunotherapy to improve therapeutic efficacy

Accumulating evidence suggests that monotherapy of surgery, radiation, chemotherapy, or even single-pronged immunotherapy is insufficient to achieve satisfactory therapeutic outcome, whereas combinational approaches that not only debunk tumors, but also elicit strong antitumor immunity during tumor debunking improve therapeutic efficacy [96,98,111,140].

Given the documented immune stimulatory property of radiotherapy, some chemotherapies, and the potential immune adjuvant function of the above described pharmalogical p53-activators, we believe that combining these therapies with active immunotherapy will further enhance the desired antitumor immunity. Because the small molecule p53-activators not only re-activate, but also reverse p53 dysfunction associated with *p53* mutations, they will provide the additional advantage of reversing p53 dysfunction-induced immune tolerance or immunosuppression of the TME to augment antitumor efficacy of active immunotherapy for tumors with both wild-type and mutant *p53*.

Certainly, radiotherapy and some chemotherapy regimens can stimulate similar levels, if not higher, of p53-activation in tumors maintaining wild-type *p53* as compared with pharmacological p53-activators. An additional immunostimulatory benefit associated with radiotherapy and chemotherapy, but not pharmacological p53-activators, is the transient lymphopenia in the host, which is important for adoptive T cell transferred based immunotherapy as it enhances T cell activation and expansion [98]. Moreover, it also has been shown that low doses of cyclophosphamide selectively suppresses the immunoinhibitory regulatory T cells, thereby enhancing antitumor immunity [98], which has not been seen in p53-pharmacological activators.

Therefore, pharmacological p53-activators and radio-/chemotherapy mediated p53-activation each has their own unique advantages and associated drawbacks. Their immunostimulatory potency in combination with active immunotherapy and translational potential will not been known until tested side-by-side. It is very likely that the selection of one approach over another will be tumor and case-specific, depending on the integrity of the p53 pathway and the TME. More in depth comparative studies are warranted before their broader clinical applications as an adjuvant for tumor immunotherapies.

## Conclusions

Despite more than 30 years of extensive studies on p53 with more than 60,000 publications, our understanding of the complexity of p53 pathway and its regulation in many biological processes is far from complete. It is indisputable that p53 suppresses tumorigenesis via the canonical pathway of inducing apoptosis and/or senescence and the non-canonical pathways, some of which are still emerging. Conversely, *p53* dysfunction-induced tumorigenesis is mediated by loss of cell cycle arrest and apoptosis, as well as by compromising host immune surveillance and altering tumor milieu to pro-tumor inflammation. This immune regulatory function of p53 is particularly exciting for tumor immunotherapy as p53-reactivation and restoration is no longer the sole molecular biology approach for cancer treatment. Instead, targeting the p53 activity and pathway, either via conventional chemotherapy and radiotherapy or the novel pharmacological activators, will prove to be more clinically important as they provide dual therapeutic effects of direct p53-activation/restoration mediated tumor killing and enhanced immune activation to promote antitumor immunity. More importantly, they can be used in combination with other active immunotherapies to maximize ultimate antitumor efficacy for tumors maintaining wild-type p53 and incurring *p53* mutations. Therefore, a better understanding of how p53 activity and of specific mechanisms/pathways that may ultimately revert tumor-induced immune tolerance to heightened immune activation is clinically significant for improving therapeutic outcome.

## Abbreviations

Trp53: p53, Transformation-related protein 53, tumor suppressor p53
TAA: Tumor associated antigen
TME: Tumor microenvironment
MDM2: Mouse double minute 2 homolog
SCO2: Synthesis of cytochrome c oxidase
TIGAR: TP53-induced glycolysis and apoptosis regulator
DRAM: Damage regulated autophagy mediator
AMPK: AMP-activated protein kinase
EMT: Epithelial to mesenchymal transition
CAF: Cancer associated fibroblasts
AICD: Activation-induced cell death
MDSC: Myeloid derived suppressor cell
STAT: Signal transducers and activators of transcription
HMGB1: High-mobility group box 1
ICD: Immunogenic cell death
IRF: Interferon regulatory factor
ISGF: Interferon stimulated gene factor
RITA: Reactivation of p53 and induction of tumor cell apoptosis
PRIMA-1: P53 reactivation and induction of massive apoptosis-1
MIRA-3: Mutant p53 reactivation and induction of rapid apoptosis
